# Lead shielding efficiency from the gamma background measurements in the salt cavern of the Polkowice–Sieroszowice copper mine

**DOI:** 10.1007/s10967-015-4567-6

**Published:** 2015-10-27

**Authors:** Kinga Polaczek-Grelik, Jan Kisiel, Agata Walencik-Łata, Jerzy W. Mietelski, Paweł Janowski, Małgorzata Harańczyk, Jan Jurkowski, Agnieszka Zalewska, Jan Kobziński, Paweł Markowski, Andrzej Sadowski

**Affiliations:** Institute of Physics, University of Silesia, Uniwersytecka 4, 40-007 Katowice, Poland; IFJ PAN, Radzikowskiego 152, 31-342 Kraków, Poland; AGH University of Science and Technology, Mickiewicza 30, 30-059 Kraków, Poland; Polkowice–Sieroszowice Mine, Division of KGHM Polska Miedź S.A., Kaźmierzów, Poland

**Keywords:** Gamma-ray spectrometry, Gamma background, Underground laboratory, Natural radioactivity, Lead shielding efficiency

## Abstract

The studies of lead shielding efficiency from the gamma background measurements were performed in the salt cavern of the copper mine - a site considered for an underground laboratory. Within the energy range of 50–2700 keV, the measured gamma-ray count rates normalized to the mass of the high-purity detectors germanium crystal are 5.93 and 6.32 s^−1^kg^−1^ for the used low-background and portable spectrometers, respectively. The gamma-ray flux of 0.124 (2) cm^−2^s^−1^ connected with the natural radioisotopes was observed by the portable HPGe, including 0.026 (1) cm^−2^s^−1^ contribution of radon decay products, whereas the photon flux at the spectrum continuum was 0.18 (5) cm^−2^s^−1^.

## Introduction

The measurements of very rare events, such as neutrinoless double beta decay, dark matter or nucleon decay searches, require extremely low background. Therefore, they are performed in the underground physics laboratories, where the rock overburden reduces substantially cosmic radiation [1, 2]. For instance, the muon flux is reduced by about 6 orders of magnitude at the depth of 2700 m.w.e [3]. However, the neutrons originating from (*α*, *n*) and the spontaneous fission reactions of U and Th rock contamination together with neutrons from residual cosmic-ray muon interactions are the source of a rather complicated and unavoidable background [4]. The neutrons produce some effects in gamma-ray spectra, although they are detectable only when the main sources of gamma-ray background are reduced, resulting in the low continuous component of gamma-ray background spectrum.

The origin of gamma-ray background spectrum is complex. In the case of the studied conditions one can distinguish among several sources of gamma-ray emitters. The first one is due the gamma emitters from the salt. The second one is connected with radon daughters present in the air inside the cavern. The third one is due to the radionuclides present inside the detector crystal, cryostat, preamplifiers etc. The use of any kind of external detector’s shielding reduces the first two components (coming from the outside of the spectrometer system), whereas the third one remains. Moreover, the shield introduces the fourth component to the background signal—the background caused by radionuclides inherent to the shield itself.

The aim of the study was to get the first empiric estimation of achievable gamma-ray background for our newly constructed low-background gamma spectrometer after its localization within the lead shield inside the P1 cavern in the Polkowice–Sieroszowice mine, KGHM Polska Miedź S.A. Holding. A similar estimation can be obtained in the course of Monte Carlo simulations; however, one has to make assumptions about the details of the geological surrounding of the cavern. The present estimation will be used to verify such simulations, which are planned for the future.

In this paper the results of in situ gamma background measurements performed in the P1 salt cavern in the Polkowice–Sieroszowice copper mine are presented. Two detectors were used: unshielded low-background HPGe (high purity germanium) detector recently constructed at the IFJ PAN, Kraków, Poland, and portable coaxial HPGe detector with reverse cathode (REGe), Canberra Industries Inc. Within the limitations of our experiment it was possible to take underground spectra only with light (2.5 cm thick) not complete lead shield (collimator) for the portable detector. Finally, the estimation of the predicted count rates expected for some gamma-ray lines for our low-background detector shielded by 10 cm of lead are presented. Using two spectrometers with comparable detector parameters (mass, efficiency, housing, see: Experimental apparatus section) but different cryostat geometry (vertical dipstick vs. horizontal) and cryostat radioisotopic purity it was possible to better characterize the contribution of particular components of gamma radiation background.

The P1 cavern is located 930 m below the surface (2200 m.w.e.), in the salt deposit, which is about 70 m thick and surrounded by anhydrite layers. The P1 cavern is 100 m long, 20 m wide and 15 m high. The salt layer is considered as a possible location of an underground physics laboratory, the SUNLAB project. A more detailed description of the Polkowice–Sieroszowice mine can be found in [5]. The results of the measurements of natural radioactivity (the radioactive isotopes concentration, radon concentration and dose) performed in 2005 in the P1 salt cavern are presented in [6].

## Experimental apparatus

Two coaxial semiconductor spectrometers were used: a low-background high-purity germanium (HPGe) detector and a commercially available portable in situ HPGe spectrometer. Additionally, the radon concentration was determined during the gamma radiation spectrum collection. This measurement was carried out about 50 cm above the salt ground for 30 h with the use of a portable detector Radon Alpha Detector (RAD-7^TM^, Durridge, USA), which is an active monitoring system based on passivated ion-implanted silicon detector (PIPS).

### Low-background laboratory spectrometer

The n-type HPGe detector manufactured at IFJ PAN from Umicore germanium monocrystal (about 600 g, 56 mm diameter, 53 mm height, 25 % relative efficiency, 2.1 keV FWHM at 1.33 MeV ^60^Co line) was placed into a low background vertical (dipstick) cryostat produced by Baltic Scientific Instruments (Riga, Latvia). The total uranium and thorium concentration in the copper elements of cryostat (e.g. cold finger) is below 0.1 ppb, whereas in the aluminium alloys of the detector holder or endcap it is below 1 ppb. The cold finger and preamplifier housings are made of stainless steel. The endcap (83 mm in diameter) is equipped with a carbon fibre composite window 0.8 mm thick and 50 mm in diameter. The preamplifier is located more than 10 cm from the bottom of the endcap. The spectrometer operates with standard Canberra NIM modules: HV supply and amplifier, and Polish MCA Tukan 8K USB (NCNR, Świerk, Poland).

This set-up was transferred to the Polkowice–Sieroszowice P1 salt cavern at the beginning of July 2014, together with the commercial portable spectrometer described below. The one-day (exactly 24 h) background spectrum of unshielded spectrometer was collected. The background of the same spectrometer at the ground level was previously characterized during the 48-day long measurement and therefore it was already well known.

### Portable in situ system

The GR4020 spectrometer (Canberra Industries, Inc., USA) consists of a reverse-electrode type high-purity germanium (REGe) detector with a crystal of 40 % efficiency, 61 mm in diameter and 63 mm in length, a portable spectroscopy workstation InSpector^TM^ 2000 (8194 channels) based on a digital signal processing (DSP^TM^) technology and a portable personal computer (PC). The detector as operating in the temperature of liquid nitrogen (LN_2_) is characterized by the resolution of 2.08 keV and the Peak-to-Compton (P/C) ratio of 57/1 at 1.33  MeV ^60^Co line. The applied spectrometric gain 5.0 together with the carbon composite entrance window (0.6 mm thick) makes possible to register gamma-rays in the energy range of 6 keV–3.2 MeV. The registration and analysis of the spectra were performed with the use of Genie^TM^ 2000 v.3.2.1 software package. The efficiency calibration has been done for the geometry of a room/box with internal surface contamination using In situ Counting mathematical Software (ISOCS^TM^). This tool utilizes the full factory characterization of a specific detector done by the manufacturer with the use of NIST-traceable sources and MCNP Monte Carlo modelling code. The dimensions of the room as well as the detector set-up corresponded to the experimental conditions. However, the distribution of radioactivity was very simplified, assuming only surface contamination instead of a real volumetric board of radionuclides. For energetic calibration the following solid sealed sources (1 cm in diameter, activity ~ 40 kBq), placed 10 cm from the detector front surface, were used: ^22^Na, ^54^Mn, ^57^Co, ^60^Co, ^65^Zn, ^109^Cd, ^133^Ba and ^137^Cs.

The detector was located approximately in the centre of the cavern, directly on the salt surface. Therefore, the germanium crystal was approximately 10 cm above the level of the cavern’s floor and directed parallel to it, i.e. facing the far end of the cavern. The spectrum was registered twice. The first spectrum was acquired for 21 h with a detector surrounded by a circular, 2.5 cm thick, lead shielding. The second spectrum (23 h) was taken using the same set-up, but with the unshielded detector.

## Results and discussion

**Fig. 1:**
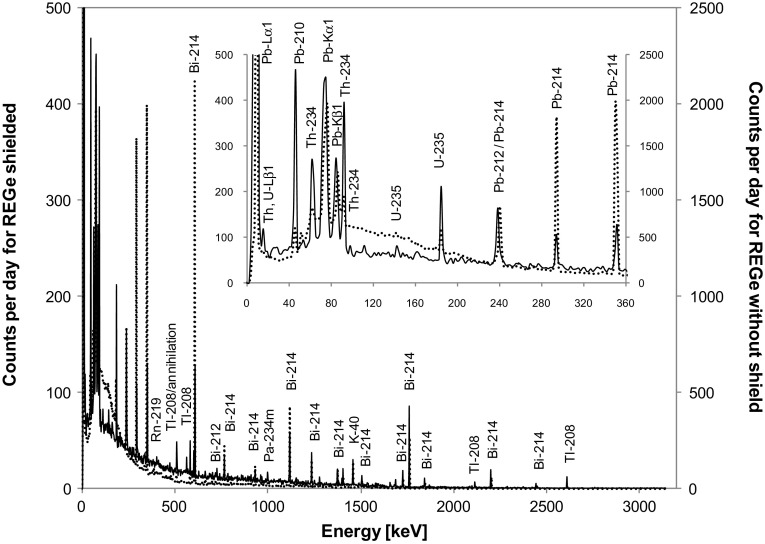
In situ gamma-ray spectra registered in the P1 cavern by the portable spectrometer (REGe) with 2.5 cm lead shielding (*solid line*) and without shield (*dotted line*). The *inset* shows a zoom of the low-energy part of these spectra up to 360 keV. The scaling factor of vertical axes corresponds to the ratio of ^40^K peak counts (i.e.~×5) for unshielded versus shielded detector

**Fig. 2:**
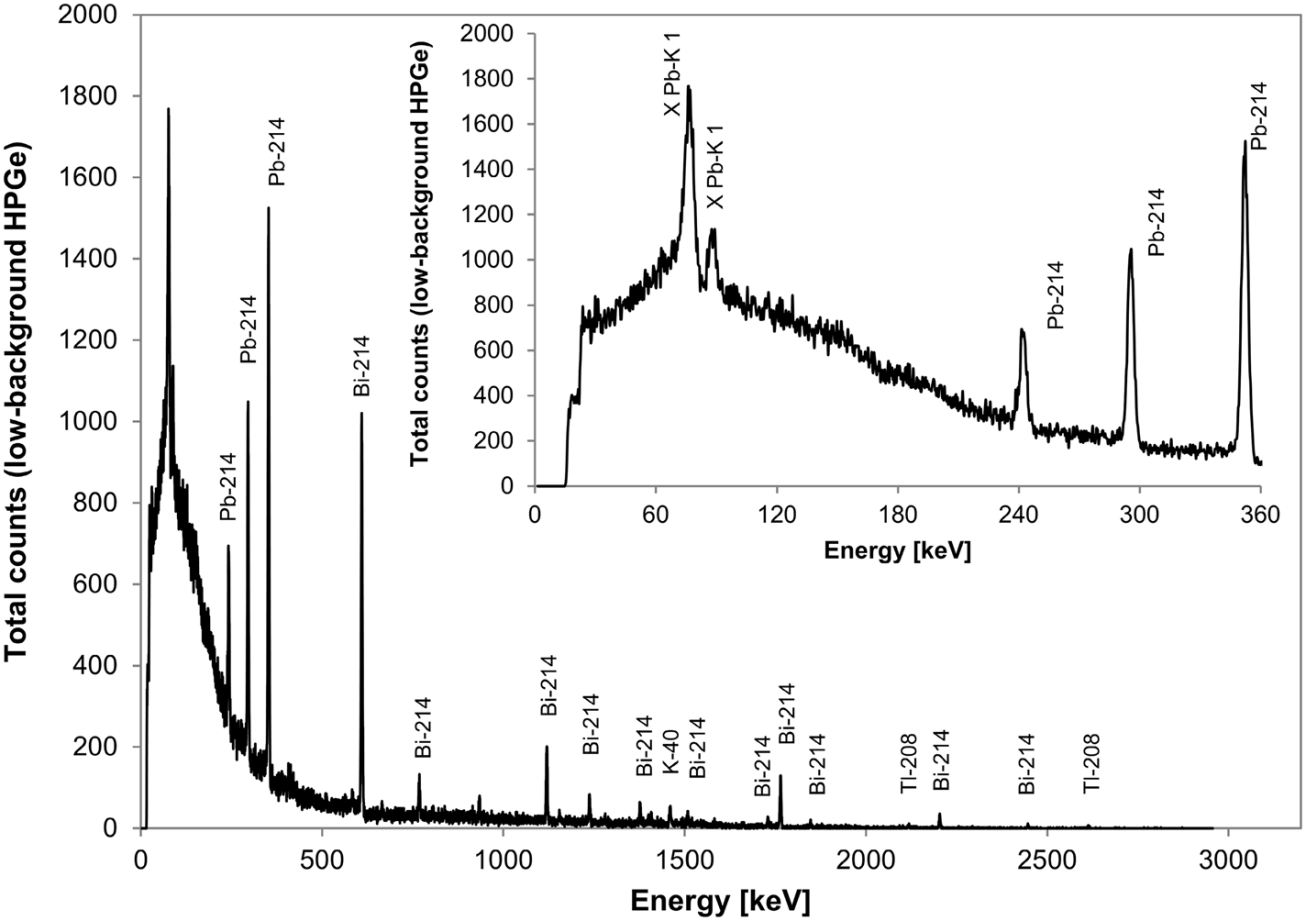
In situ gamma-ray spectrum registered for 24 h in the P1 cavern by the low-background HPGe spectrometer without shielding. The *inset* shows a zoom of the low-energy part of the spectrum up to 360 keV

The obtained spectra of gamma radiation registered in the centre of the salt cavern using the portable REGe detector with 2.5 cm-thick lead shielding and without it as well as for the unshielded low-background spectrometer are presented in Figs. 1 and 2, respectively. The spectra clearly demonstrate that the studied location has surprisingly low potassium content. The other important property of this location is the lack of annihilation peak on the spectrum of low-background spectrometer. This part of the gamma-ray spectrum is usually assigned to the cosmic component [7, 8] in underground laboratories. Moreover, the spectrum of unshielded low-background spectrometer (Fig. 2) is dominated by the decay products of ^222^Rn. The lack of 186 keV ^226^Ra photopeak indicates that it is airborne radon, transported with the ventilation ducts, and not originating onsite from ^226^Ra incorporated in the salt.

The detailed identification of photopeaks and the results of their quantitative analysis, together with the values of the statistical uncertainties (*k* = 1), for both detectors are given in Table 1.

**Table 1:** Naturally occurring radionuclides registered in the P1 cavern with the spectrometric systems used: unshielded low-background HPGe and unshielded as well as shielded (2.5 cm Pb) portable HPGe

Decay chain	Isotope	Energy (keV)	Count rate (s^−1^)		
			Portable HPGe		Low-background HPGe
			Unshielded	With 2.5 cm Pb shield	
–	X Pb	10.55	0.3126 (23)	0.5029 (26)	–
Uranium	^210^Pb	46.54	0.0219 (12)	0.0270 (8)	<0.02
Uranium	^234^Th	63.29	0.0159 (17)	0.0160 (9)	<0.0063
Uranium	^234^Pa	73.92	–	0.0153 (6)	<0.0074
–	X Pb	74.97	0.0611 (12)	0.0304 (7)	<0.0074
–	X Bi	77.12	0.1041 (14)	0.0073 (5)	<0.0072
–	X Pb	84.94	–	0.0142 (5)	0.0185 (18)
–	X Bi	87.34	0.0343 (10)	0.0057 (4)	<0.0063
Uranium	^234^Th	92.59	0.0238 (10)	0.0192 (10)	<0.0058
Actinium + Uranium	^235^U + ^226^Ra	185.71 + 186.21	0.0158 (15)	0.0108 (6)	<0.0043
Thorium	^212^Pb	238.63	0.0121 (6)	0.0092 (4)	<0.0043
Actinium	^219^Rn	271.23	–	0.0044 (2)	<0.0039
Uranium	^214^Pb	295.21	0.1082 (14)	0.0043 (6)	0.0577 (12)
Uranium	^214^Pb	351.92	0.1820 (17)	0.0075 (6)	0.0957 (13)
Actinium	^219^Rn	401.81	–	0.0039 (2)	<0.0021
Thorium	^208^Tl	510.77	0.0038 (6)	0.0028 (4)	<0.0016
Thorium	^208^Tl	583.19	0.0033 (4)	0.0021 (3)	0.0017 (6)
Uranium	^214^Bi	609.31	0.1549 (14)	0.0125 (4)	0.0687 (10)
Uranium	^214^Bi	665.45	0.0042 (5)	0.0002 (2)	0.0014 (3)
Thorium	^212^Bi	727.33	0.0012 (2)	0.0005 (2)	<0.0012
Uranium	^214^Bi	768.36	0.0142 (6)	0.0023 (3)	0.0059 (4)
Uranium	^214^Bi	964.77	0.0013 (2)	0.0003 (2)	<0.00096
Uranium	^234m^Pa	1001.03	0.0014 (4)	0.0008 (2)	<0.0010
Uranium	^214^Bi	1120.29	0.0328 (7)	0.0055 (4)	0.0129 (5)
Uranium	^214^Bi	1238.11	0.0121 (5)	0.0024 (2)	0.0045 (3)
–	^40^K	1460.83	0.0107 (4)	0.0023 (2)	0.0036 (3)
Uranium	^214^Bi	1764.50	0.0246 (6)	0.0068 (3)	0.0090 (4)
Uranium	^214^Bi	2204.21	0.0060 (3)	0.0017 (2)	0.0024 (2)
Thorium	^208^Tl	2614.53	0.0015 (1)	0.0012 (1)	0.00033 (6)

Only the lines registered at least on two spectra are presented

The comparison of the data presented in Table 1 shows that for the nuclides of thorium and actinium series the count rates of the shielded and unshielded portable detector are close to each other, especially at low energies, i.e. the shielding does not reduce substantially photons coming from actinium and thorium series, although the observed decrease is beyond the uncertainty level. This confirms that the detector at low energies mainly counts photons which are produced within the cryostat or germanium crystal itself, i.e. caused by radioactive impurities of the detector. Moreover, the uranium/actinium/thorium ratio seen by a standard high-purity germanium (REGe) portable spectrometer is equal to 0.619 (13)/0.016 (2)/0.022 (2) for the unshielded detector and 0.133 (6)/0.019 (1)/0.016 (1) with the shielding applied.

In the case of the uranium series one can also notice that the count rates ratio between the shielded and unshielded portable detector is changing with energy, but for low energies the values are still higher than they should be in the case of the absence of internal detector impurities. This demonstrates that the uranium series nuclides are inside the detector. The count rate within the potassium ^40^K line matches the efficiency of the lead shielding used, which suggests that the measured potassium is the component of the salt rather than constituting the internal detector impurity in this case.

**Fig. 3:**
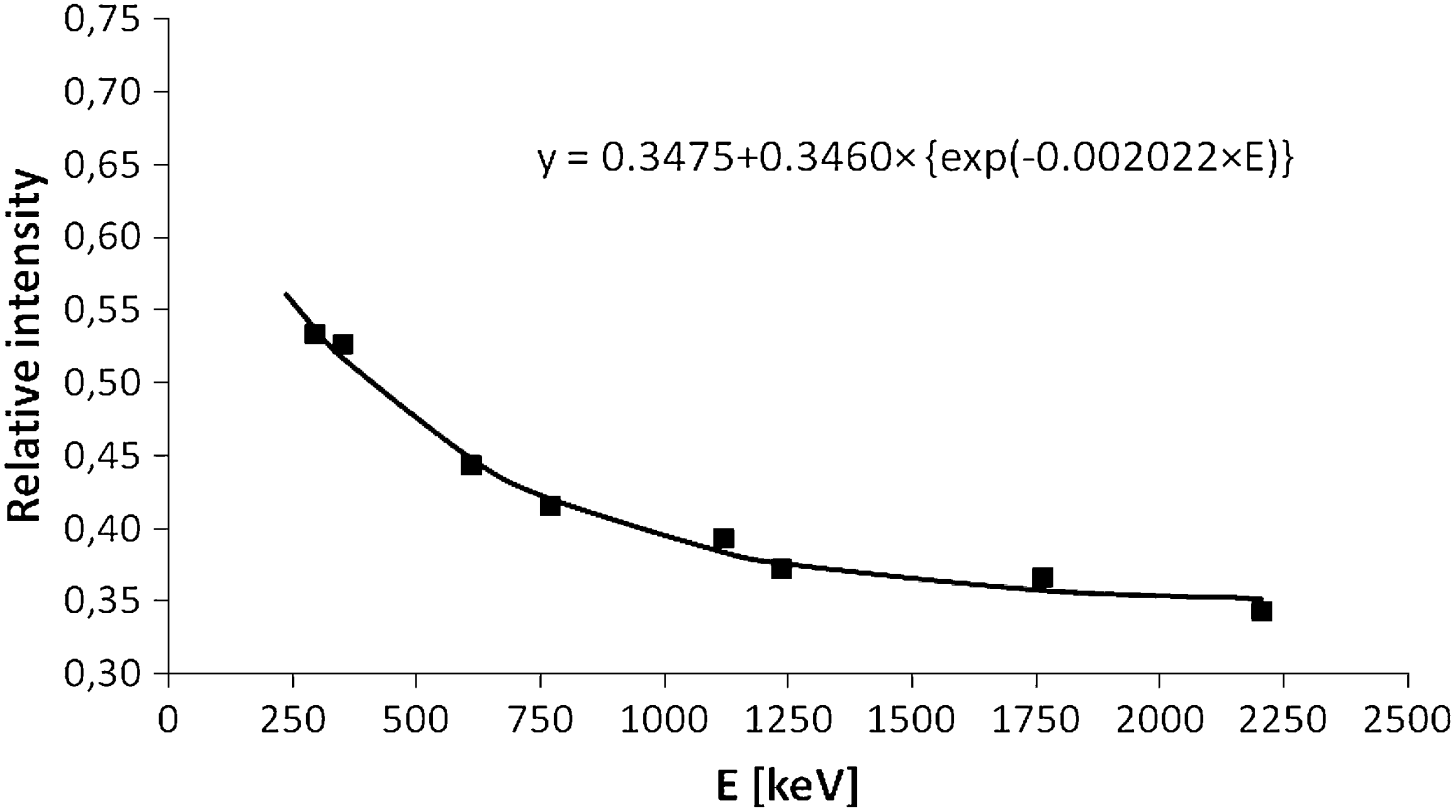
The ratio between the count rates of the unshielded low background (HPGe) detector and portable (REGe) detector for main gamma lines (cps_HPGe_/cps_REGe_) = f(E) from uranium series members (^214^Pb and ^214^Bi). The count rates were measured in the P1 salt cavern

The ratio between the count rates for the gamma lines of uranium member series for both unshielded detectors used can be described by an exponential curve with constant fraction (Fig. 3). For higher energies it becomes constant and equal to 0.35. In the case of an absence of internal impurities in the portable detector, the ratio plot in Fig. 3 should be ≈1 regardless the energy, since the counting efficiency for both detectors used are close to each other. The ratio <1 indicates that more radioimpurities is present in the portable detector than in a low-background one, what was also concluded before on the basis of data presented in Table 1. We have found that also for ^40^K this ratio is about 0.34. However, for thorium series, namely from ^208^Tl at 2615 keV, it is equal to 0.22. This in turn confirms that the majority of gamma photons from the decay of thorium series originates from the portable detector impurities, and therefore for a low background system we can expect lower values.

The comparison of the gamma-ray flux in several underground laboratories, presented in Table 2, demonstrates that the investigated location exhibits an extremely low level of natural radiation, both measured with the low-background spectrometer and registered by the portable detector. In this table, for comparison purpose, our earlier result [6] is also shown, obtained with the use of detector with different housing, i.e. not equipped with the carbon composite window.

**Table 2:** The comparison of the integral count rates in the energy range of 7–2734 keV in several European underground experimental sites, as seen by the HPGe detectors

Location	Boulby	Gran Sasso	Modane	Sieroszowice		
				Portable REGe spectrometer	Low-background HPGe spectrometer	Portable HPGe spectrometer
Count rate (s^−1^)	6.5–28 (several locations)	8–60 (several locations)	15–108 (several locations)	5.79 (1)	3.92 (1)	1.95 (2)^a^
Source	[9]	[10]	[11]	This study	This study	[6]
Detector type	HPGe, p-type	HPGe, p-type	HPGe, p-type	HPGe, n-type	HPGe, n-type	HPGe, p-type
Relative efficiency (%)	32	32	32	40	25	30

^a^Different measuring range (40–2700 keV) and detector endcap (without carbon-composite entrance window)

On the base of the photo-peak count rates registered by the REGe detector and by applying the efficiency determined by ISOCS^TM^, the gamma-ray flux has been determined as 0.124 (2) photons per cm^2^ per second. However, this value seems to be overestimated since the efficiency obtained using mathematical calibration software (ISOCS^TM^) is probably underestimated, i.e. achieved from the model of radioactivity distribution on the cavern surface instead of being uniformly distributed within the volume of the salt deposit. The main radon daughters: ^210^Pb,^214^Pb and ^214^Bi, are responsible for 0.026 (1) cm^−2^s^−1^ photon flux, whereas the internal impurities of portable spectrometer setup (inherent detector and shielding radioimpurities) have the main impact on the registered gamma radiation with this system of about 67 %. If the radioactive impurities connected with experimental setup were suppressed (the case of low-background spectrometer), the main background contribution would be connected with radon decay products, whereas potassium ^40^K salt-content contribution would be about 3 %.

The total spectrum continuum photon flux reaches 0.18 (5) cm^−2^s^−1^. This value is a superposition of photons emitted by radionuclides, scattered inside the detector volume and electromagnetic avalanche connected with cosmic radiation and its secondaries. Therefore it should not be directly referenced to the contribution of muon-related events.

**Fig. 4:**
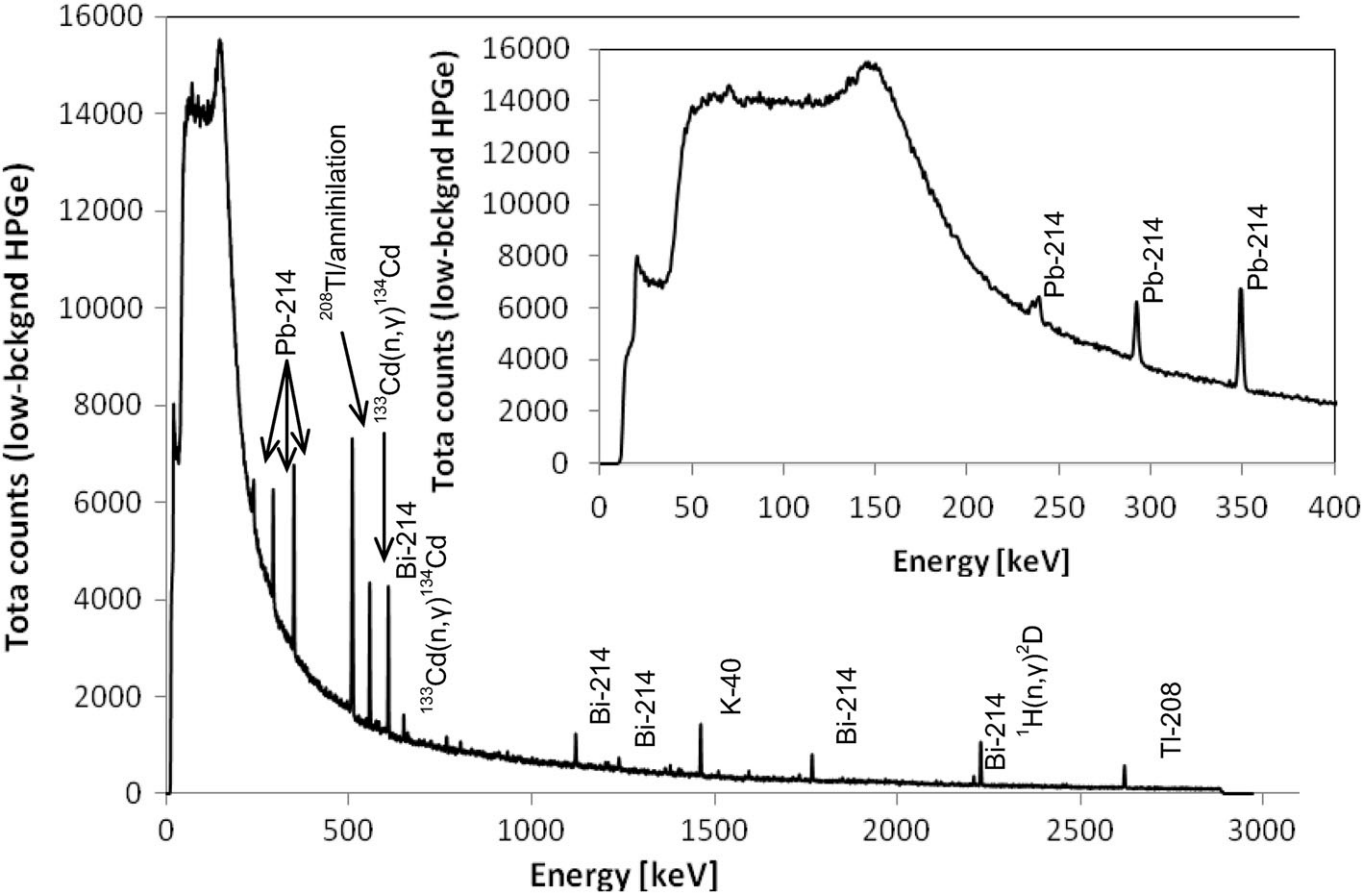
Gamma-ray spectrum registered for 48 days at the ground level in the IFJ PAN by the low-background HPGe spectrometer with 10 cm lead shielding. The aim of this measurement, beside the background study, was to increase neutron effects, thus the shield included also water moderator (50 L) placed around the detector and 2 mm thick cadmium plate placed from its top. The *inset* in the graph shows a zoom of the low-energy part of the spectrum up to 400 keV

The comparison with the spectrum registered on the earth’s surface by the low-background detector (shown in Fig. 4), placed within the housing of lead 10 cm thick, has been performed. One can notice that for energies below 600 keV the *d* = 10 cm lead shield reduces external gamma-rays to a negligible level, namely the attenuation factor exp(μd), based on linear attenuation coefficient μ, is higher than 10^6^ [12]. Thus, from the low energy part of the spectrum it is possible to estimate the effective count rates from the internal (detector and shield) content of radionuclides and then correct the high energy part, finally calculating the expected effective count rate. The constructed low-background HPGe spectrometer is ultimately planned to operate in the salt layer in configuration with a 10 cm lead housing. In the P1 cavern in the simplified approach one can measure the count rates for different lines in the unshielded spectrometer and scale them by attenuation coefficients for 10 cm lead, which for example results in scaling factors of 7.44 × 10^−7^ for 609 keV line and of 0.0077 for 2614 keV line. The spectra registered with the shielded and unshielded portable system (Fig. 1) can be used to demonstrate that such an approach is misleading, which is due to the non-scaling component of the intrinsic impurities. However, the results obtained with the portable detector can be used to estimate the effective reducing power for the shield made of standard lead in the case of ^40^K and ^208^Tl, after taking into consideration the correction to standard portable detector radioactive impurities. Such an assessment could be then applied to estimate the reducing power of the planned 10 cm thick lead shield. For example, the reduction factor of the gamma-ray flux for a fourfold increase of lead thickness (from 2.5 to 10 cm) is equal to e^4^ ≈ 55. However, such a simple approach cannot be applied since the low-background detector has its own radioactive non-scalable impurities. In the case of the estimation of the dominating gamma emitter—^222^Rn decay products, we took under consideration that radon levels are similar on the ground level [5, 13, 14] and in the P1 cavern and equal to 15.4 ± 1.1 Bq/m^3^, as measurements done in 30-hour cycle have shown. For energies below 700 keV (namely: 295, 352, 609 keV) we assumed that the count rates (as governed by the radioactive impurities within the cryostat, detector and in-shield radon daughters levels) on the ground level and in the P1 cavern would be the same. As mentioned above, the 10 cm lead shield is thick enough to eliminate any outer sources for low energy gammas, i.e. the spectrometer background is caused only by radioactivity within the shielded volume. Then we calculated the excess of count rates for 768, 1120, 1238 and 1764 keV lines for the ground level laboratory, taking into account the count rates from low energy peaks and using a calibration curve for cylindrical geometry. In our prediction, the intensities of radon-daughters lines are taken from the ground level background measurement for the shielded detector reduced by this factor. A similar reduction of “excess” was used for the calculations of the predicted count rate for ^208^Tl in 2614 keV line on the basis of 911 keV line of ^228^Ac. For potassium we assumed conservatively the same count rate underground as for the ground level. The results are presented in Table 3.

**Table 3:** Count rates (s^−1^) for the main lines for low-background gamma-rays spectrometer: shielded by 10 cm of lead, obtained in 48-day measurements on the ground level; unshielded, measured underground in the P1 salt cavern; expected for the shielded by 10 cm of lead in P1 salt cavern

Energy (keV)	Origin [3, 4]	Ground level, shielded		Unshielded, P1 cavern (the same as in Table 1)	Prediction for shielded in P1 cavern^a^
		(s^−1^)	Relative uncertainty		
242.0	^214^Pb	0.001248	0.075		0.0012
295.2	^214^Pb	0.002114	0.039	0.0587	0.0021
351.1	^214^Pb	0.003709	0.020	0.0957	0.0037
510.8	e^+^e^−^	0.007602	0.0098		<0.0001
609.3	^214^Bi	0.002902	0.018	0.0687	0.0029
768.4	^214^Bi	0.000288	0.12	0.0059	0.00027
911.2	^228^Ac	0.000142	0.27		0.00013
1120.3	^214^Bi	0.000682	0.051	0.0129	0.00060
1238.1	^214^Bi	0.000293	0.11	0.045	0.00022
1460.8	^40^K	0.001222	0.026	0.0036	0.0012
1764.5	^214^Bi	0.000572	0.043	0.0090	0.00036
2614.3	^208^Tl	0.000625	0.033	0.00033	0.00010

^a^See text for details

## Conclusions

Measurements performed with the use of shielded and unshielded configuration of two gamma spectrometry systems clearly demonstrated that there is a difference in the gamma-ray count rates coming from particular radioactive decay series and registered by semiconductor spectrometers placed in the middle of the underground salt cavern. The use of two spectrometers (commercial and low-background) with comparable dimensions and crystal housings allowed to confirm that thorium and actinium series isotopes can be effectively suppressed by careful choosing of a detector material.

The comparison of the spectra registered by the commercially available portable REGe detector and the specially constructed low-background HPGe spectrometer demonstrates that internal radioactive impurities within the measurement device in the environment with a very low-level of natural radioactivity are the main sources of the background signal influencing the registered spectra. Under some approximate assumption the prediction of the efficiency of a lead shielding (of the thickness being in agreement with the numerical findings [15]) was made. According to these calculations, with the studied low-background spectrometer in the shielded (10 cm Pb) configuration the background reduction of about 30 % could be achieved.

Moreover, the influence of the reduction of internal radioimpurities/background gamma-radiation signal, achieved by the construction of low-background detector in comparison with standard portable systems, can be roughly assessed from the ratio (cps_HPGe_/cps_REGe_) = f(E) developed on the base of uranium series and expand to other nuclides in high-energy region, to test their internal—detector or external—environmental origin.

It has been shown (Table 2) that the obtained results are dependent from the type of germanium crystal, i.e. the n-type spectrometer register lower count rates than p-type systems. Therefore, they could not be related straightforward to each other, although other features of the detectors are comparable.

Unlike the underground laboratories located in different geological formations (e.g. [16]), the radon concentration in the salt chamber of copper mine in Poland is comparable to the ground level concentration, being of the order of 15 (1) Bq/m^3^. This confirms that the main source of radon and its products is the ventilation air and not the radium incorporated in the salt rock. The obtained result of radon concentration is in agreement with literature reported investigations for salt mine locations, e.g. INR Solotvina laboratory [3], Khewra Salt Mines [14].

Among the advantages of studied location in deep underground salt mine (2200 m w.e.) low levels of radon concentration and ^40^K count rate (0.004 s^−1^kg^−1^) as well as the undetectable level of cosmic muon component (annihilation peak) on the spectrum of low-background spectrometer should be stressed. Therefore, these findings support the salt layer of the Polkowice–Sieroszowice mine as the location of a low-background underground laboratory—the SUNLAB (Sieroszowice UNderground LABoratory) project.
